# Patient mistreatment, social sharing of negative events and emotional exhaustion among Chinese nurses: the combined moderating effect of organizational support and trait resilience

**DOI:** 10.1186/s12912-024-01924-x

**Published:** 2024-04-22

**Authors:** Wei Yan, Xiu Chen, Di Xiao, Huan Wang, Xin Du, Li Li, Chunjuan Xu, Caiping Song

**Affiliations:** 1 School of Economics and Business Administration, Chongqing University, Chongqing, China; 2 Medical Insurance Office, Hospital of Chongqing University, Chongqing, China; 3 School of Medicine and Health Management, Tongji Medical College, Huazhong University of Science & Technology, Wuhan, China; 4 Development and Planning Department, Chongqing Medical University, Chongqing, China; 5 Medical Center of Hematology, Xinqiao Hospital, State Key Laboratory of Trauma, Burn and Combined Injury, Army Medical University, Chongqing, China; 6 Human Resources Department, The Second Affiliated Hospital of Chongqing Medical University, Chongqing, China; 7 Department of Burn Plastic and Microsurgery, The No. 987 Hospital of Joint Logistic Support Force of PLA, Baoji, China; 8 Xinqiao Hospital, Army Medical University, No. 83 Xinqiao Main Street, Shapingba District, Chongqing, China

**Keywords:** Patient mistreatment, Emotional exhaustion, Social sharing of negative events, Organizational support, Resilience trait, Combined effects

## Abstract

**Background:**

As a primary form of work-related violence in the healthcare sector, patient mistreatment negatively impacts nurses’ well-being. To date, there has yet reached a definitive conclusion on the mediating mechanism and boundary conditions behind the influence of patient mistreatment on nurses’ emotional exhaustion.

**Methods:**

This study employed a convenience sampling method to recruit a sample of 1672 nurses from public hospitals in Western China. The data were collected through anonymous self-report questionnaires and analyzed using hierarchical regression and conditional processes to investigate a theoretical framework encompassing patient mistreatment, emotional exhaustion, social sharing of negative events, organizational support, and trait resilience.

**Results:**

Patient mistreatment led to emotional exhaustion among nurses (β = 0.625, *p* <.001), and social sharing of negative events mediated this positive relationship (effect = 0.073, SE = 0.013). The combined effects of organizational support and resilience moderated the mediating effect of the social sharing of negative events between patient mistreatment and emotional exhaustion (β=-0.051, *p* <.05). Specifically, nurses with a high level of resilience would benefit from organizational support to alleviate emotional exhaustion caused by patient mistreatment.

**Conclusions:**

This study validated a significant positive association between patient mistreatment and emotional exhaustion, which aligns with previous research findings. Integrating conservation of resources theory and goal progress theory, we addressed previous contradictory findings on the impact of social sharing of negative events on emotional exhaustion. Social sharing of negative events served as a mediator between patient mistreatment and emotional exhaustion. Additionally, the moderating effect of organizational support on the relationship between social sharing of negative events and emotional exhaustion depended on individual trait of resilience.

## Introduction

Workplace violence is a worldwide concern and a major risk in healthcare work. It was defined as incidents in which staff members are mistreated, threatened, or assaulted in circumstances related to their work [1]. Over the last decades, it has been well documented that healthcare professionals around the world are at significant risk of violence exposure [2]. Studies have shown that the most vulnerable healthcare workers victimized are nurses and paramedics [3, 4], with the most common perpetrators being patients, their relatives, or visitors [5]. A recent survey of 4263 nurses in the healthcare sector showed that 54% of respondents had experienced verbal violence by patients [6], including negative emotional behaviors exhibited by patients or their families, such as anger, swearing, insults, yelling, and speaking rudely toward nurses [7–9]. All these negative emotional behaviors are known as “patient mistreatment”. A considerable amount of research conducted in healthcare organizations has shown that exposure to patient mistreatment is a strong predictor of stress, emotional exhaustion, turnover intention and obstacles to career development among nurses [6, 7, 10–13]. Specifically, emotional exhaustion, characterized by intense fatigue, lack of interest, low mood, and less enthusiasm for jobs, is not only a key outcome resulting from patient mistreatment but also serves as a significant predictor of nurse turnover and a decline in nursing job performance. The conservation of resources (COR) theory provides a theoretical framework for understanding emotional exhaustion caused by patient mistreatment. The COR theory indicates that people strive to retain, protect, and build resources which are needed in fulfilling job responsibilities and are threatened by the potential or actual loss of those valued resources [14–16]. Despite increasing research interest, the existing literature has yet reached a definitive conclusion on the mechanism how patient mistreatment impacts nurses’ emotional exhaustion. Therefore, this study developed and examined a theoretical model regarding the influence of patient mistreatment on nurses’ emotional exhaustion and explored the mechanisms and boundary conditions behind this relationship.

Social sharing of negative events refers to talking to others about negative events and one’s emotional reactions to them and can occur hours to months after the event [17, 18]. It is often seen as a response to emotional experiences to release negative emotions, alleviate work-related stress, and restore resources. However, there is no consensus on the impact of this behavior on emotional exhaustion [19–21]. Social sharing of negative events sometimes fails to bring new insights into emotional experiences, and disrupts nurses’ goal-related cognitive processes. Goal progress theory illustrates that goal failure (e.g. receiving customer mistreatment) [22] is associated with cognitive rumination [23], which may lead to the further loss of resources. Therefore, we examined the social sharing of negative events as a mediating mechanism in the relationship between patient mistreatment and emotional exhaustion in this study.

Furthermore, studies of organizational support have shown that it provides a supportive environment for individuals in coping with stress caused by customer mistreatment [24]. The COR theory also explicates that supportive environments and contexts create fertile ground for creation of individual resources [15]. However, some evidence has revealed that organizational support is not consistently beneficial, yielding inconclusive findings [25–27]. Besides, it is crucial to understand why some people are able to handle negative experiences at work more functionally than others. Consistent with COR theory, individual resources may be contained or embodied in traits and capabilities [14]. Resilience is a personal trait that can help individuals better cope with adversity and stress [28]. Therefore, this study introduces organizational support as a crucial moderating variable to explore its moderating effect on the mediating pathway of the social sharing of negative events between patient mistreatment and emotional exhaustion and examines whether trait resilience serves as a boundary condition to the effectiveness of organizational support.

In summary, drawing upon the conservation of resources theory and goal progress theory, this study attempts to answer the following questions: Is patient mistreatment related to emotional exhaustion through the social sharing of negative events? Is organizational support always beneficial or not? And who will benefit from it?

### Patient mistreatment and emotional exhaustion

Among all occupational groups, healthcare workers are ranked as one of the most likely to experience workplace aggression [29–32]. Patient mistreatment refers to negative emotional behaviors such as expressed anger, swearing, insulting, yelling, and speaking rudely directed toward healthcare providers by patients or their families [9, 33, 34]. Existing studies have extensively explored the adverse consequences of patient mistreatment on healthcare staff and found that it can negatively impact their psychological and physical well-being, leading to increased anxiety, burnout, and negative emotions [35–37]. The psychological harm caused by patient mistreatment can also result in stress, which is defined as a reaction to an environment in which there is a threat or net loss of resources [34].

The conservation of resources (COR) theory constructs a framework for comprehending the origins and coping strategies of stress and is frequently used to interpret the process of emotional exhaustion. Individual resources are defined as any element that is valuable for an individual’s survival and development. Individuals strive to retain, protect, and build the resources they value [14–16], and suffer salient impacts when they lose resources. Moreover, the availability of resources determines the impact of workplace stressors (such as unfair treatment) on employees [38, 39]. Healthcare professionals may experience emotional exhaustion, which refers to energy depletion or the draining of emotional resources [38], as a consequence of mistreatment by patients [35]. Therefore, we propose the following hypothesis:

#### H1

Patient mistreatment is positively related to emotional exhaustion.

### The mediating role of social sharing of negative events

Researchers have identified social sharing of negative events as talking to others about a negative event and one’s emotional reactions to it and can occur hours to months after the event [17, 18, 40]. Individuals voluntarily share their negative emotional experiences and feelings with others in social settings to release negative emotions, alleviate work-related stress, and restore psychological resources. Despite research on this topic, there is no consensus on the impact of social sharing on negative emotions. Delroisse et al. suggested that it can reduce job burnout by helping employees make sense of work situations and reinforcing relationships with others [19]. By contrast, Nolen-Hoeksema posited that sharing could potentially be detrimental if it involves ruminating on or immersing oneself in negative feelings, potentially exacerbating or prolonging feelings of sadness [20]. Drawing upon the conservation of resources theory and goal progress theory, we aimed to clarify the effect of social sharing of negative events between patient mistreatment and emotional exhaustion.

COR theory stated that individuals should proactively invest resources to protect themselves against potentially stressful situations, recover from losses, and accumulate additional resources to brace themselves for future challenges [14–16]. Social sharing of negative events has been conceptualized as a social and interpersonal process of repetitively seeking proactive social opportunities to verbalize experiences of stressful events [40, 41]. Strongman et al. argued that social sharing of emotions activates the interconnectedness between individuals and their respective social networks or support systems [42]. Supportive actions by recipients, such as listening, understanding, and consolation, help sharers replenish depleted resources and foster their ability to cope with stressors in the sharing process [43], ultimately equipping them with the necessary resources to address adverse situations. For example, Zech highlighted that social sharing of negative events can provide informational support (e.g. advice) and facilitate reevaluation for individuals [17]. Laurens’s study revealed that nurses are inclined to engage in emotional social sharing with professionals, such as colleagues or counselors, when confronted with emotional issues involving their patients [44]. Therefore, drawing upon conservation resources theory, we anticipated that nurses who experience resource depletion due to patient mistreatment may seek to obtain the necessary resources through social sharing of negative events to manage stressful events.

Social sharing of negative events can facilitate cognitive-affective processing of shared events [45]. However, it carries “sharing risks” [46], particularly when negative emotions are involved. When it comes to repeated negative events, deliberate thoughts oriented towards the implications of a given event may alternate with unwanted, intrusive thoughts [40]. Martin and Tesser defined a class of conscious thoughts that revolve around a common instrumental theme as cognitive rumination [23], which is associated with goal progress theory [22] to illustrate the impact of goal failure (e.g., receiving customer mistreatment) [47]. Patient mistreatment serves as a pivotal emotional event and an original disruption. It fails to bring new insights into emotional experiences, disrupts nurses’ goal-related cognitive processes, and triggers rumination [40, 47] when nurses share negative events with others [20]. The more nurses ruminate, the longer they experience intrusive thoughts linked to unachieved goals [22]. Moreover, loss of resources or the threat of such loss is a crucial factor in predicting psychological distress and leading to investing more resources, making those already lacking resources even more vulnerable to loss spirals [14]. Emotional exhaustion occurs when individuals are confronted with dual stressors of resource depletion and goal failure. Consequently, we propose the following hypothesis:

#### H2

Social sharing of negative events mediates the relationship between patient mistreatment and emotional exhaustion.

### The moderating role of organizational support

Hobfoll et al. further clarified those resources, which are central to survival and goal attainment, operate depending on the ecological context [48, 49].They further theorized that resources do not exist individually but travel in packs, or caravans for both individuals and organizations [15, 50]. Organizational support, which is the overall belief that the organization values contributions and cares about the well-being of its employees [51], is a vital aspect of work resources. Crossover acts as one of the mechanisms of resource exchange within resource caravans [15] and states that organizational support can be effectively transferred from organizational context to individuals. Studies have suggested that the crossover of resources is also very important for gaining spirals because it can increase a partner’s engagement, potentially triggering a chain of crossover of engagement processes [52]. Moreover, global research has also identified organizational support as a new buffer-type resource that can counter the resource-depleting effect of high workload and high emotional demands in a large sample of Dutch health professionals [53]. Therefore, these important work resources, including concern, recognition, and respect inherited in organizational support, would compensate for individuals’ resources, foster the accomplishment of personal work objectives [54], and enhance employees’ self-efficacy and sense of self-worth, consequently elevating their positive emotions [55, 56]. Thus, we anticipated that organizational support would not only alleviate the adverse effects of mistreatment experienced by employees within the organization [57–59], but also effectively moderate the relationship between social sharing of negative events and emotional exhaustion.

### The combined effect of organizational support and trait relicense

Conventionally, studies have demonstrated that organizational support constitutes a valuable work resource. However, COR theory posited that the transfer of resources across social entities (individuals and organizations) is slower. Mounting evidence suggested that organizational support may, at times, not be helpful or even worsen situations [60–62]. Perhaps the effects of crossover depend on certain traits of the individuals or groups. Evidence continued to mount regarding those with greater resources being less vulnerable to resource loss and more capable of gaining resources [15, 63]. Luthans and Avolio [64] pointed out that both psychological capital and organizational support are necessary for employees to achieve high performance. Resilience, an individual’s ability to cope effectively with adversity and stress when facing difficulties and setbacks [65, 66], can be a key personal resource for understanding how individuals break loss spirals [67, 68]. Resilience enables individuals to adapt better to changing environments [69, 70] and shapes their perception of stress [71, 72].

This study found that trait resilience acts as a boundary condition for the moderating role of organizational support in the relationship between social sharing of negative events and emotional exhaustion. Furthermore, the interactive effects among various resources, such as psychological and organizational resources [73], do not simply add up, but rather enhance the assets necessary for individuals to accomplish their objectives. Consequently, it facilitates individuals with higher levels of resilience by employing both personal psychological resources and organizational resources to develop effective strategies to handle challenges like patient mistreatment [74]. In conclusion, this study proposes the following hypotheses:

#### H3

The moderating effect of organizational support on the relationship between social sharing of negative events and emotional exhaustion depends on trait resilience.

#### H4

 The interaction between organizational support and trait resilience moderates the indirect effect of patient mistreatment on emotional exhaustion via the social sharing of negative work events.

**Fig. 1:**
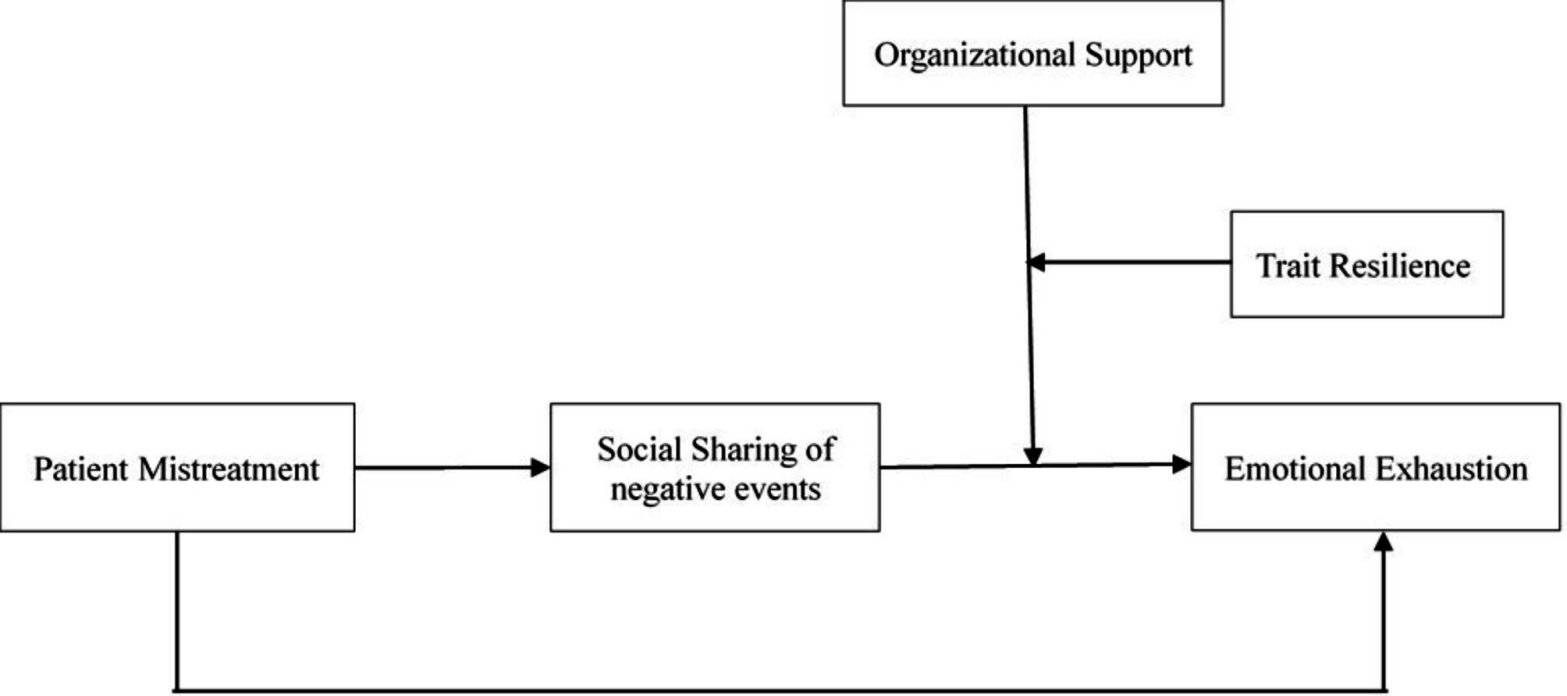
Hypothesized theoretical model

We summarize our theoretical model in Fig. 1.

## Method

### Participants and data collection procedures

Convenience sampling was employed in this study. We initiated a call for nursing mistreatment research based on the Hematology Specialty Alliance platform in Chongqing, a major city in Southwest China. Furthermore, we used one-on-one communication to invite the clinical department nurses to participate in the survey. The inclusion criteria for recruiting participants in our study were as follows:① Certified nurses; ② Clinical nursing positions; ③ Informed consent and voluntary participation. The exclusion criteria were as follows:① student nurses in rotation, ② student nurses on internships, ③ nursing residents in training programs, and④ off-duty nurses (on leave, sick leave, or attending external training).

To minimize the risks posed by the COVID-19 pandemic, this study employed a structured online questionnaire to facilitate ease of participation. To ensure the credibility and fairness of the collected data, all responses were submitted anonymously. The questionnaires were completed anonymously to ensure the acquisition of objective and unbiased data. The initial page of the questionnaire presented a clear statement of the study’s objectives and confidentiality of the responses. All questions were designed to be mandatory, and each unique IP address was allowed a single submission to uphold the integrity of the data and avoid duplicate entries. In preparation for the main study, a preliminary survey was conducted to validate the logic of the questions and the accuracy of their responses. The formal survey was conducted from October 9th, 2022 to November 1st, 2022. (Questionnaire link: https://wj.qq.com/mine.html), ultimately yielding 1627 valid responses.)

### Measures

We employed the translation and back-translation processes recommended by Brislin [75] in both surveys prior to the administration. This was done to ensure the validity and appropriateness of all the scales in the Chinese context.

#### Patient mistreatment

We used the 18-item scale developed by Wang et al. [21] to measure patient mistreatment, replacing the word “customers” with “patients” in each item. The scale divides patient mistreatment into two dimensions: aggressive mistreatment and demand-oriented mistreatment. Participants rated the items on a five-point Likert scale from 1 = never to 5 = frequently. Example items were “Patients demanded special treatment,” “Patients spoke aggressively to you,” and “Patients asked you to do things even if they can do them themselves.” The Cronbach’s alpha of the scale was 0.953.

#### Social sharing of negative events

Social sharing of negative events scale was adapted from Gable et al. [76]. In the past month, participants were asked how often they had talked to significant others, other family members, friends, and colleagues about unpleasant things that had happened at work, creating a four-item scale. Responses ranged from 1 = never to 5 = often. Cronbach’s α coefficient was 0.862.

#### Emotional exhaustion

Emotional exhaustion was measured using the Chinese version of the Maslach Burnout Inventory (MBI), which was developed by Maslach and Jackson [77] and is the most widely used tool for evaluating job burnout. Emotional exhaustion included nine items, with sample items such as, “I feel emotionally drained from my work.” Responses ranged from 1 = strongly disagree to 5 = strongly agree. All the items scored positively, with higher scores indicating greater emotional exhaustion. Cronbach’s α coefficient was 0.925.

#### Organizational support

 In this study, we employed the Organizational Support Perception Scale originally developed and validated by Shen and Benson in 2016 [78] to assess the perceptions of organizational support. This scale consists of eight items (e.g. “My organization values my contributions to the organization”) and used a 7-point Likert scale. Among these items, four were positively worded and four were reverse-scored. Respondents indicated their agreement on a scale ranging from 1 = strongly disagree to 7 = strongly agree, with higher scores indicating a stronger perception of organizational support. Cronbach’s alpha for the scale was 0.907.

#### Resilience

We used the Brief Resilience Scale (BRS) developed by Smith et al. [79], which consists of six items. Sample items included statements such as “I tend to bounce back quickly after difficulties.” Responses ranged from 1 = strongly disagree to 5 = strongly agree. Three items scored positively and three scored negatively. It is specifically used to measure an individual’s ability to recover their health or well-being in response to stress. Cronbach’s α coefficient was 0.826.

#### Control variables

Sex, age, education, marital status, years of work, and sports were included as control variables to control for confounding effects on emotional exhaustion.

### Data analysis

SPSS23.0 and Mplus7 were used for the statistical analysis. We adopted confirmatory factor analysis to test validity and common method variance. Additionally, we conducted a descriptive statistical analysis of the variables and analyzed each variable using the Pearson’s correlation test to comprehend the characteristics and correlations between the variables. We performed hierarchical regression analysis and conditional process analysis to examine the mediating and moderating effects. Moderating variables were mean-centered to construct the interaction term, mitigating potential multi-collinearity problems. In this study, patient mistreatment served as a predictor variable, social sharing of negative events as a mediator variable, organizational support and resilience as two moderators, and emotional exhaustion as the outcome variable.

## Results

### Participants

A total of 1627 valid responses were included after a strict review of the collected survey data. The majority of the participants were female (94.7%), while males accounted for only 5.3% of the sample, which is similar to the composition of nurses in other public hospitals in China. Most nurses (87.7%) were between 20 and 39 years old, with two under 20 years old, and 6.9% were over 40 years old. The participants’ years of work experience ranged from less than one year to 36 years, with an average of 9.26 years (SD = 6.40). The majority of nurses (62.6%) were married, and only 36.5% of the total participants reported exercise habits.

### Common method variance

Data collected from a single source require querying for possible interference caused by common method variance (CMV). Harman’s single-factor method was used to detect the common method variance. The results of the exploratory factor analysis of the 45 items showed that there were seven factors with eigenvalues greater than 1, and the variance explanation rate of the first factor was 31.579% (< 50%). Therefore, the results suggested that CMV is not a significant problem in this study [80, 81].

### Confirmatory factor analysis

We conducted confirmatory factor analysis (CFA) to assess the discriminant validity of the scale. As shown in Table 1, the five-factor model, consisting of patient mistreatment, social sharing of negative events, organizational support, resilience, and emotional exhaustion, demonstrated satisfactory discriminant validity and good fit (χ²/df = 11.276, RMSEA = 0.079, CFI = 0.819, TL = 0.809, SRMR = 0.057). Each variable had a factor loading greater than 0.600 and the internal consistency was good, indicating satisfactory reliability and validity of the scale.

**Table 1:** Confirmatory factor analysis results

Model	χ^2^	df	χ^2^/df	RMSEA	CFI	TLI	SRMR
five-factor model (PM,SS,OS,RE,EE)	10543.337	935	11.276^***^	0.079	0.819	0.809	0.057
four-factor model (PM,SS,OS + RE,EE)	12254.790	939	13.051^***^	0.086	0.787	0.776	0.063
three-factor model (PM,OS + RE,SS + EE)	15057.331	942	15.984^***^	0.096	0.735	0.721	0.076
two-factor model (PM + OS + RE,SS + EE)	22594.396	944	23.935^***^	0.119	0.593	0.573	0.140
one-factor model (PM + OS + RE + SS + EE)	29943.802	945	31.686^***^	0.137	0.455	0.429	0.155

*Notes*:*n* = 1627; ***p <.001;

PM, patient mistreatment; EE, emotional exhaustion; SS, social sharing of negative events; OS, organizational support; RE, resilience

### Descriptive statistics

Table 2 presents the means, standard deviations, and correlation coefficients for the variables used in this study. The correlation coefficients were consistent with our expectations, showing that patient mistreatment was significantly positively correlated with emotional exhaustion (*r* =.361, *p* <.01) and with the social sharing of negative events (*r* =.198, *p* <.01). Additionally, the social sharing of negative events was positively correlated with emotional exhaustion (*r* =.253, *p* <.01). Some of the hypotheses of this study were tentatively supported.

**Table 2:** Descriptive statistics and correlations

Variables	Mean	SD	1	2	3	4	5
**1.PM**	3.06	0.75	0.953				
**2.EE**	1.26	1.34	0.361^**^	0.862			
**3.SS**	2.85	1.03	0.198^**^	0.253	0.925	.	
**4.OS**	3.82	1.08	-319^**^	− 0.471^**^	− 0.193^**^	0.907	
**5.RE**	3.52	0.67	− 0.218^**^	− 0.484^**^	− 0.192^**^	0.520^**^	0.926
.							

*Notes*: *n* = 1627; When applicable, alpha reliability coefficients are presented on the diagonal

*p <.05. **p <.01;. PM, patient mistreatment; EE, emotional exhaustion; SS, social sharing of negative events;

OS, organizational support; RE, resilience

Patient mistreatment: Items are rated on a five-point Likert scale from 1 = never to 5 = frequently

Social sharing of negative events: Items are rated on a five-point Likert scale from 1 = never to 5 = often

Emotional exhaustion: Items are rated on a five-point Likert scale 1 = strongly disagree to 5 = strongly agree

Organizational support: Items are rated on a seven-point Likert scale from 1 = strongly disagree to 7 = strongly agree

Resilience: Items are rated on a five-point Likert scale from 1 = strongly disagree to 7 = strongly agree

### The mediating role of social sharing of negative events

Hierarchical regression was used to test the relevant hypotheses and the results are presented in Table 3. Model 4 indicated a positive correlation between patient mistreatment and emotional exhaustion (β = 0.625, *p* <.001), which supported Hypothesis 1. The test for the mediating effect followed the recommended stepwise approach [82]. First, Model 2 revealed a significant positive correlation between patient mistreatment and the social sharing of negative events (β = 0.275, *p* <.001). Second, Model 5 showed that social sharing of negative events was positively correlated with emotional exhaustion (β = 0.264, *p* <.001). Finally, while the effect of patient mistreatment on the dependent variable, emotional exhaustion, remained significant (β = 0.552, *p* <.001), it was somewhat weaker (0.552 < 0.625) after introducing the mediating variable, suggesting a partial mediating effect.

**Table 3:** The mediating role of social sharing on the relationship between patient mistreatment and emotional exhaustion

	Social sharing of negative events				Emotional exhaustion					
	Model 1		Model 2		Model 3		Model 4		Model 5	
	β	t	β	t	β	t	β	t	β	t
Gender	0.276^*^	2.349	0.305^**^	2.649	0.118	0.793	0.184	1.323	0.104	0.761
Age	− 0.010	− 0.768	− 0.003	− 0.268	− 0.029	-1.743	− 0.014	− 0.899	− 0.013	− 0.861
Education	− 0.023	− 0.285	− 0.024	− 0.305	0.111	1.092	0.109	1.143	0.115	1.239
Marital status 2	0.119	1.769	0.103	1.560	− 0.266^**^	-3.124	− 0.303^***^	-3.805	− 0.330^***^	-4.243
Marital status 3	− 0.074	− 0.387	− 0.094	− 0.503	− 0.459	-1.891	− 0.505^**^	-2.229	− 0.480^*^	-2.171
Marital status 4	-1.192	-1.609	-1.263	-1.738	1.932^*^	2.053	1.773^**^	2.018	2.106^**^	2.454
Working years	0.010	0.803	0.006	0.502	− 0.003	− 0.162	− 0.011	− 0.769	− 0.013	− 0.899
Sport	− 0.026	− 0.552	− 0.009	− 0.191	− 0.290^***^	-4.801	− 0.251^***^	-4.440	− 0.248^***^	-4.505
Patient mistreatment			0.275^***^	8.243			0.625^***^	15.474	0.552^***^	13.723
Social sharing of negative events									0.264^***^	8.993
R²	0.012		0.052		0.063		0.184		0.223	
F	2.463^*^		9.829^***^		13.538^***^		40.411^***^		46.254^***^	
ΔR²	0.012		0.040^*^		0.063^*^		0.121^*^		0.039^*^	
f²	0.012		0.042		0.063		0.638		0.050	

*Note* *p <.05, **p <.01, ***p <.001 (two-tailed)

Gender: 1 = male, 2 = female, Marital status 2 = married, Marital status 3 = divorced, Marital status 4 = widowed

Following Preacher and Hayes [83], this study further tested the mediating effect of the social sharing of negative events on the relationship between patient mistreatment and emotional exhaustion. We employed the bias-corrected method with a sample size of 5000 and a 95% confidence interval to perform multiple mediating effect analysis using Process3.2, a software for conditional process analysis. The test results are presented in Table 4. The results showed that the indirect effect was 0.073, with a 95% confidence interval of [0.049, 0.100], demonstrating that the social sharing of negative events played a mediating role in the relationship between patient mistreatment and emotional exhaustion. Therefore, H2 was supported.

**Table 4:** Bootstrap test for mediating effect

Mediator variable	Effect	SE	95%CI		*p*
			LLCI	ULCI	
Mediating effect: Patient mistreatment→Social sharing of negative events→Emotional exhaustion					
Social sharing of negative events	0.073	0.013	0.049	0.100	0.000

### The combined moderating effect of organizational support and trait resilience

**Fig. 2:**
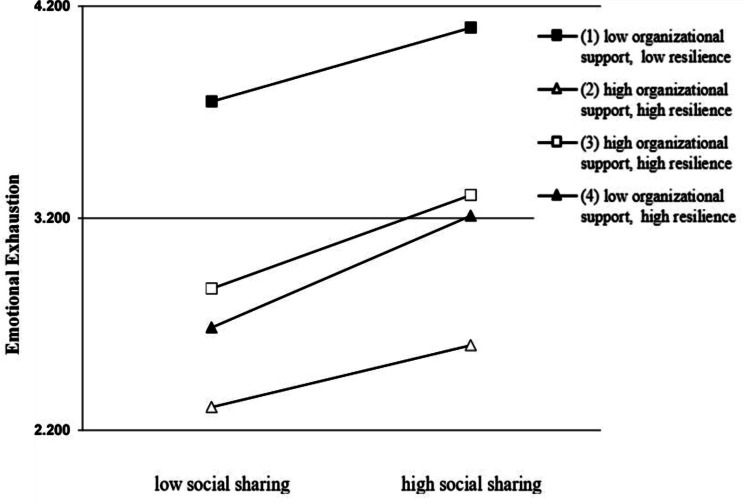
Simple slope test

Table 5 presents the results of moderation analysis. In Model 2, both organizational support and resilience were found to be significantly negatively correlated with emotional exhaustion (βos=-0.348, *p* <.001; βre = − 0.569, *p* <.001). However, in Model 3, neither organizational support nor resilience showed any interaction with social sharing of negative events in predicting emotional exhaustion. Nevertheless, the three-way interaction between social sharing of negative events, organizational support, and resilience was significant in predicting emotional exhaustion and negatively correlated with emotional exhaustion (β=-0.051, *p* <.05), thus supporting H3. Figure 2 shows the results of the three-way interaction, in which it is evident that higher levels of organizational support and resilience weaken the positive impact of the social sharing of negative events on emotional exhaustion.

**Table 5:** The combined effect of organizational support and resilience on the relationship between social sharing of negative events and emotional exhaustion

	Emotional exhaustion							
	Model 1		Model 2		Model 3		Model 4	
	β	t	β	t	β	t	β	t
Gender	0.023	0.160	− 0.045	− 0.361	− 0.058	− 0.468	− 0.059	− 0.474
Age	− 0.025	-1.594	− 0.010	− 0.754	− 0.011	− 0.776	− 0.010	− 0.723
Education	0.119	1.215	0.048	0.572	0.037	0.435	0.041	0.482
Marital status 2	− 0.307^***^	-3.741	− 0.276 ^***^	-3.905	− 0.268^***^	-3.801	− 0.274^***^	-3.883
Marital status 3	− 0.433	-1.855	− 0.446^**^	-2.213	− 0.431^**^	-2.151	− 0.437^**^	-2.180
Marital status 4	2.343^**^	2.585	1.477	1.886	1.508	1.935	1.480	1.899
Working years	− 0.006	− 0.395	− 0.010	− 0.775	− 0.009	− 0.712	− 0.009	− 0.730
Sport	− 0.281^***^	-4.831	− 0.161^***^	-3.182	− 0.155^**^	-3.090	− 0.159^**^	-3.160
Social sharing	0.345^***^	11.353	0.202^***^	7.505	0.207^***^	7.705	0.229^***^	7.877
Organizational support			− 0.348^***^	-11.779	− 0.362^***^	-12.128	− 0.372^***^	-12.298
Resilience			− 0.569^***^	-11.899	-598^***^	-12.387	− 0.621^***^	-12.522
Social sharing of negative events *Organizational support					− 0.017	− 0.647	− 0.011	− 0.414
Social sharing of negative events *Resilience					− 0.015	− 0.348	− 0.006	− 0.140
Organizational support*Resilience					0.121^***^	3.548	0.133^***^	3.845
Social sharing of negative events *Organizational support* Resilience							− 0.051^*^	-1.972
R²	0.132		0.355		0.362		0.364	
F	27.307^***^		80.670^***^		65.411^***^		61.419^***^	
ΔR²	0.132^*^		0.223^**^		0.008		0.002	
f²	0.132		0.346		0.011		0.003	

*Note* β represents the standardized regression coefficients for each step in the regression equation

*p <.05, **p <.01, ***p <.001 (two-tailed)

Gender: 1 = male, 2 = female, Marital status 2= married, Marital status 3= divorced, Marital status 4= widowed

We also conducted a moderated mediation model in Process 3.2, using 95% bias-corrected bootstrap confidence interval analyses with 5,000 bootstrap samples to examine the moderating effect of the interaction term of organizational support and resilience on the mediating role of social sharing of negative events between patient mistreatment and emotional exhaustion. As shown in Table 6, the index of moderated moderated mediation was − 0.0152, which was statistically significant, with a 95% bias-corrected confidence interval of [-0.0286, − 0.0031]. Therefore, H4 was supported.

**Table 6:** Indices of conditional moderated mediation by organizational support

Moderator variable	Index	SE	95%CI	
			LLCI	ULCI
Low resilience (-1 SD)	0.0057	0.0093	-0.0120	0.0240
Resilience (Mean)	-0.0044	0.0070	-0.0181	0.0091
High resilience (+1 SD)	-0.0146	0.0070	-0.0291	-0.0012
Index of moderated moderated mediation	-0.0152	0.0065	-0.0286	-0.0031

Specifically, the 95% confidence interval for the indices of conditional moderated mediation was [-0.0120, 0.0240] for individuals with high resilience and [-0.0291, -0.0012] for those with low resilience. Therefore, H3 was supported, indicating that individual resilience served as a boundary condition for the moderating effect of organizational support on the relationship between the social sharing of negative events and emotional exhaustion.

## Discussion

This study combined conservation of resources theory with goal progress theory to investigate the mediating role of the social sharing of negative events in the association between patient mistreatment and nurses’ emotional exhaustion. We also explored whether the moderating effect of organizational support on the relationship between the social sharing of negative events and emotional exhaustion depended on individual resilience. First, this study confirmed a significant positive correlation between nurses’ experiences of patient mistreatment and emotional exhaustion, which is consistent with previous studies [6, 7, 84–86]. The findings once again underscore the detrimental impact of patient mistreatment on nurses’ emotional and psychological well-being. Given that the rates of different forms and sources of aggression vary considerably between nations [3, 87], it is crucial to direct our attention towards the patient mistreatment experiences of nurses in China, especially in the post-epidemic era.

Second, this study revealed that the social sharing behavior of negative events mediates the relationship between patient mistreatment and emotional exhaustion. Previous studies have produced mixed findings regarding the impact of the social sharing of negative events on emotional exhaustion among employees or nurses [19–21]. However, limited research has examined the role of social sharing of negative emotions as a mediating mechanism between patient mistreatment and nurses’ emotional exhaustion. This study integrated the conservation of resources theory and goal progress theory to establish a theoretical foundation for the mediating model. It indicated that sharing negative work events was a strategy for nurses to cope with resource loss resulting from patient mistreatment. Meanwhile, rumination about negative events was closely associated with goal failure, thereby triggering emotional exhaustion among nurses.

Third, the interaction between resilience and organizational support served as a moderator in the relationship between the social sharing of negative events and emotional exhaustion. Studies have identified organizational support as a crucial resource for mitigating the negative effects of stressors [24]. However, our findings demonstrated that there was no significant two-way interaction between social sharing of negative events and organizational support in predicting emotional exhaustion. This finding is in line with some research on organizational support [25, 28], which suggested that organizational support may fail to alleviate the adverse effects of work stressors. Furthermore, this study responded to the call for conservation resources theory [28] to explore whether trait resilience serves as a boundary to the effectiveness of organizational support. The significant three-way interaction between the social sharing of negative events, organizational support, and trait resilience revealed that individuals with high levels of resilience will benefit from organizational support. Specifically, individuals with high resilience and organizational support showed lower levels of emotional exhaustion than those with low resilience and high organizational support. The implication for managers, therefore, was that organizational support alone cannot solve all problems. Instead, individualized organizational support should be considered in the light of nurses’ resilience.

### Practical implications

The findings of this study have significant practical implications for medical management. First, the findings of this study once again validated the significant influence of patient mistreatment on nurses’ emotional exhaustion. Consequently, it is imperative for healthcare administrators to prioritize the establishment of a secure working environment for nurses while providing comprehensive training programs that could enhance their ability to react more effectively to navigate complex nurse-patient relationships. Second, the study further showed that the social sharing of negative events predicted emotional exhaustion among nurses. Therefore, finding ways to eliminate negative rumination originating from patient mistreatment is essential for reducing emotional exhaustion among nurses. Mindfulness thinking, meditation or psychological detachment from work are potential means that nurses could adopt to take a different perspective on negative events. Although the current study indicates that organizational support may not always be beneficial, we suggest that management consider developing workplace interventions that facilitate supportive relationships between organizations and nurses. Third, it is noted that the effect of organizational support depended on resilience. Resilience-related training programs may help nurses acquire psychological resources, enabling them to effectively navigate through mistreatment and adverse experiences. For instance, professional provider resilience training (PPRT) conducted by the medical department of the US military provides knowledge and skills to assist in stress management [88], such as developing positive cognition, emotional regulation, and mind-body techniques, which enhances the psychological resilience of medical professionals and alleviates fatigue and burnout.

### Limitations and further study

This study has some limitations worth addressing. First, the study design was cross-sectional, which may have limited its ability to capture unexamined longitudinal associations. Thus, experience-sampling method should be employed to study the fluctuations of the relationship examined in this study on daily or week basis. Second, all variables investigated were self-reported, which may raise concerns regarding common method variance (CMV) [89]. Therefore, future studies should employ objective measures or measures reported by others to reduce same-source bias. Third, we found that the social sharing of negative events only partially mediated the relationship between patient mistreatment and emotional exhaustion. Further investigations should be conducted to explore other pathways linking patient mistreatment with nurses’ emotional exhaustion, as well as the moderating variables influencing these mediating mechanisms.

## Conclusion

This study, involving 1672 healthcare nurses from public hospitals in Western China, revealed a notable prevalence of patient mistreatment, which led to emotional exhaustion among all participants. The findings of this study suggest that the sharing of negative events plays a mediating role in the relationship of patient mistreatment and the subsequent emotional drain experienced by nurses. These results serve as a critical alert to medical managers about the profound impact of negative emotional sharing within healthcare settings. Furthermore, the study highlights the importance of valuing and fostering certain personal traits of nurses, such as resilience, which can buffer the effects of patient mistreatment on emotional exhaustion, particularly when coupled with high levels of organizational support. Consequently, it is suggested to combine a supportive organizational culture in healthcare sector with training programs that aims to enhance nurses’ resilience.

## Data Availability

Data supporting the findings of this study are available upon request from the corresponding author.
